# Drastic change in China's lakes and reservoirs over the past decades

**DOI:** 10.1038/srep06041

**Published:** 2014-08-13

**Authors:** Xiankun Yang, Xixi Lu

**Affiliations:** 1 Department of Geography, National University of Singapore, 117570, Singapore; 2 Global Change and Watershed Management Center, Yunnan University of Finance and Economics, Kunming, Yunnan, 650221, China

## Abstract

Using remote sensing images, we provided the first complete picture of freshwater bodies in mainland China. We mapped 89,700 reservoirs, covering about 26,870 km^2^ and approximately 185,000 lakes with a surface area of about 82,232 km^2^. Despite relatively small surface area, the total estimated storage capacity of reservoirs (794 km^3^) is triple that of lakes (268 km^3^). Further analysis indicates that reservoir construction has made the river systems strongly regulated: only 6% of the assessed river basins are free-flowing; 20% of assessed river basins have enough cumulative reservoir capacity to store more than the entire annual river flow. Despite the existence of 2,721 lakes greater than 1 km^2^, we found that about 50 lakes greater than km^2^ have formed on the Tibetan Plateau resulting from climate change. More than 350 lakes of ≥1 km^2^ vanished in four other major lake regions. Although the disappearance of lakes happened in the context of global climate change, it principally reflects the severe anthropogenic impacts on natural lakes, such as, the excessive plundering of water resources on the Inner Mongolia-Xinjiang Plateau and serious destruction (land reclamation and urbanization) on the eastern plains.

Large water bodies (e.g. lakes and reservoirs) have been the subject of great interest not only because of their water resources role but also as indicators of anthropogenic impact on water scape change. For example, closed lakes, which have no significant surface or subsurface outflow, are particularly sensitive to human activities and have been used in previous studies^1^,^2^,^3^. Other studies of large water bodies include the noting of both the anthropogenic and natural causes of the fluctuating surface area of large lakes and reservoirs^4^,^5^,^6^. From an economic and social point of view, Nilsson et al.^7^ reported that there are more than 45,000 large reservoirs worldwide – defined as those impounded by dams higher than 15 m – used for water supply, power generation, flood control, etc. Their economic and social significances have also been reported, for example, currently, about 20% of cultivated land worldwide is irrigated, representing approximately 300 million ha, which produces about 33% of the world's food supply. Approximately 20% of the global generation of electricity is attributable to hydropower schemes, which equates to about 7% of worldwide energy usage^5^.

In terms of the importance of large lakes and reservoirs in China (Figure 1), water resource investigation has been conducted since the 1950s, which showed 2,928 lakes with an area of greater than 1 km^2^^9^,^10^ and only 20 reservoirs with a storage capacity of greater than 0.1 cubic kilometer^11^. Over the past six decades, human activities and climate change have driven complex physical and ecological changes to China's inland water bodies. For example, China has seen a remarkable reservoir development across the country and dramatically accelerated shrinkage in natural lakes in North and Northwest China. By 2013, 98,000 reservoirs had been built^12^; but, spatial information on such changes is scattered and incomplete, making it impossible to assess the impact of human activities and climate change on decadal-scale water body changes, although the central government releases the yearbook of China water resources every year and some statistical reports^12^ have been driven based on the yearbooks.

Due to the incompleteness of information on such changes and the dire need of examination how climate change and anthropogenic activities may have affected such changes in mainland China, the objectives of this study are therefore: (a) to develop a relatively complete national inventory of lakes and reservoirs using remote sensing techniques; (b) to assess the impact of reservoirs on natural river systems; and (c) to provide a comprehensive view of decadal-scale water scape changes, which is used to investigate the causes of water scape changes.

## Results

### Quantity and surface area of delineated lakes and reservoirs

This study delineated 185,333 lakes and 89,696 reservoirs, covering 82,232 and 26,870 km^2^ of the territorial land surface, respectively (Tables 1 and 2). Overall, about 1.2% of China's territorial land surface is covered by lakes and reservoirs. This percentage is much less than the global average of approximately 2.8% reported by Downing et al.^4^. For example, for a formerly glaciated area such as Canada, Sweden and Finland, the lake distribution is much higher than the global average distribution^13^, which pushes up the global proportion. However, the contribution of reservoirs to the total surface area in China (~0.29%) is much greater than the global average (0.17%). Despite relatively small surface area, the total estimated storage capacity of reservoirs (794 km^3^) is triple that of lakes (268 km^3^). From this standpoint, it can be concluded that water resources are primarily regulated by reservoirs and anthropogenic impacts on water regulation are more severe in mainland China than global average.

Figure 2 shows the cumulative frequencies of the number of lakes and reservoirs: the smallest water bodies occur at the highest frequency, and with increasing surface area, their frequency decreases exponentially. It illustrates that most lakes and reservoirs are small: 97.2% of reservoirs and 98.4% of lakes are less than 1 km^2^ in surface area. However, large water bodies account for most nationwide water surface area. For example, only 1.6% of lakes are greater than 1 km^2^, but they account for approximately 92% of the lake surface area; the largest 22 lakes, ranging from 500 to 4,278 km^2^, have 36% of the total lake surface area. In Table 2 the higher frequency of lower-order small reservoirs indicates a greater number of opportunities for dam construction because they are comparably small, but the construction is easier and cheaper, which addresses a large number of potential needs in mainland China.

### Spatial distribution and abundance of the reservoirs

Figure 3a shows the variation of reservoir density across mainland China. It shows a clear east-to-west gradation in the spatial distribution: most of the reservoirs are located in the eastern regions such as the middle and lower Yangtze River Basin, the lower Pearl River Basin and some small river basins in Southeast China. Few reservoirs are located in China's vast western regions such as the Tibet Plateau and the Inner Mongolia and Xinjiang Plateau, indicating that reservoir construction is the combined result of naturally occurring stream morphometry phenomena and potential needs that have anthropogenic impacts. The highest density of dams occurs in the Poyang Lake floodplain in the middle Yangtze River Basin, which has a density of 73 reservoirs per 100 km^2^. The high reservoir density again reflects the legacy of this region's long history of milldams for aquaculture, irrigation and other needs.

The number and estimated storage capacity of reservoirs is shown in Table 3, which shows that the flows of most of the large rivers in China have been strongly dammed, except for the Yangtze (0.28 yr) and Pearl River (0.32 yr). Taking the Yellow River as an example, the estimated total reservoir capacity of 65 km^3^ is comparable to more than triple of the river's long-term annual water discharge of 20.7 km^3^ per year^14^. The situation is also at an alarming rate in northern China, where in areas such as the Liaohe River Basin and the Haihe River Basin, the estimated reservoir capacity is almost twice as large as the annual runoff. Because the western half of China is mostly desert or mountains, the resulting concentration of Chinese population, industry, and agriculture has been roughly equivalent to squeezing the entire U.S. population into the region east of the Mississippi, then multiplying it by five^15^. Since the 1950s, water withdrawal and consumption in China have increased by approximately 5-fold because of a doubling of the population and increased irrigation and industrial activity^16^. Rapid urbanization and high population density in these river basins are the major drivers of dam construction.

The situation in South China is relatively moderated, despite the presence of more than 43,000 dams in the Yangtze River Basin and nearly 17,000 dams in the Pearl River Basin. It would seem that dams have a relatively lower net impact on the annual water discharge in these two river basins because the water discharges at the outlet (Datong station) of the Yangtze River and other key hydrological stations on the Pearl River have not yet shown significant reduction^17^,^18^,^19^. The Three Gorges Reservoir (TGR) with a total capacity of 39.3 km^3^, the world's largest hydropower project, began to impound water in June 2003; however, its impact on the Yangtze water discharge has been minor^18^ because its storage capacity is less than 5% of the Yangtze's annual discharge. Nevertheless, dam construction has drastically altered annual and seasonal sediment discharge in the upper and lower Yangtze River Basin^20^,^21^,^22^.

### Spatial distribution and abundance of lakes

Figure 3b shows the uneven distribution of lakes in mainland China. Approximately 50% of the lakes are located on the Tibet Plateau, covering 42,423 km^2^ of the land area on the Tibet Plateau. The EPL contains approximately 25% of the lakes, most of which are shallow and eutrophic^9^. Despite the large area covered by the IMXL, only 15.3% of the lakes are distributed in this region. Among the five lake regions, EPL and TPL contain the largest number of lakes, which account for nearly 75% of the total lake area in mainland China, and form two dense lake clusters in East and West China respectively. NPML is also a relatively dense lake cluster in Northeast China. A similar trend for the lakes larger than 1 km^2^ was also observed. Of the 2,721 lakes ≥ 1 km^2^, 1,140 are located in the TPL and 601 lakes are distributed in the EPL. Although the IMXL also has nearly 600 large lakes, their total surface area (10,710 km^2^) is rather less than its counterpart in the EPL (17,980 km^2^).

The distribution of lake area in different river basins is summarized in Table 4. The estimated average lake density across mainland China is 2.02 water bodies per 100 km^2^. If these lakes were evenly distributed across mainland China, this would be equivalent to an average net catchment area of 48.5 km^2^ per lake. Overall, about 0.9% of the mainland China is covered by lakes. Lake densities range from <1 water body per 100 km^2^ in much of the south-west and north-west river basins to >7 in the flat Huaihe River Basin. Thus, on a basin-by-basin basis the flat Huaihe River Basin leads the list in Table 4; other restricted areas of high density occur in the Pearl River, Songhua, Yangtze, Haihe and some inland river basins in the Tibetan Plateau. The river basins in Northwest China have the lowest lake density.

When water surface areas in Tables 3 and 4 are examined, it is persuasive evidence that unaltered natural lakes still account for a large percentage of water surface area across mainland China. From this perspective, China's water scape is much different from that in the United States because unaltered natural lakes other than the Great Lakes account for only a small percentage of water area across the conterminous United States^6^. However, it should be noted that the lake dataset in this study also includes many regulated lakes; they were identified as ‘lakes’, apparently because of their proper names (e.g. Lake Huanggai, Lake Daye, etc.) on the auxiliary maps that assisted image interpretation.

## Discussion

### Lakes are disappearing, but reservoirs still booming

Before the foundation of the People's Republic of China in 1949, China had no more than 40 small hydroelectric dams and only a handful of large-scale reservoirs; since then, the reservoir construction experienced fast development. The two decades of the 1950s and 1960s saw the addition of nearly 72,000 reservoirs in China, including approximately 280 large ones, more than any other decades, with a relatively slow increase after the 1980s^23^ (Figure 4a). Nevertheless, some reservoirs built during the period of 1950s ~ 1970s were in poor quality, approximately 4,000 of which have been abandoned in the 1980s and 1990s, followed by more than 1,000 abandonment events due to ageing and lack of proper maintenance (Figure 4a). However, the year-by-year increase of the total reservoir storage capacity of the nation shows that the greatest rate of increase in storage capacity was after 2000 after the closures of many huge reservoirs^23^ (Figure 4b). Although the oft-heard colloquial wisdom that “the dam building era is over in developed countries” is born after 1980^24^, China's dam building, especially huge dams (see Figure 4b), still booms nowadays.

The information on numbers and size and the total area of lakes in different regions obtained in this study were compared with equivalent information provided by previous studies^9^,^10^,^25^. Previous studies on China's lakes were carried out primarily based on digitized maps with scales of 1:100 000 (30-m resolution) and some topographic maps with scales of 1:50 000 (15-m resolution). Because of similar spatial resolution, this study result is directly comparable to previous research results. However, to decrease the effect of possible errors, the comparison focuses on only large lakes with surface area greater than 1 km^2^. Figure 5 shows the contrasts of number and surface area of lakes (≥1 km^2^) between our results and data documented by Wang and Dou^9^. It shows that the total number of lakes of ≥1 km^2^ declined from 3,026 in the period of the 1950s–70s to 2,848 in the 1980s, further to 2,721 at present. The total lake area decreased from 91,290 km^2^ to 80,645 in the 1980s, further to 73,139 km^2^, a loss of about 20% of total surface area over the past 60 years.

It should be highlighted that most of the large Chinese freshwater lakes are regulated to meet the needs of agricultural irrigation, flood protection and industrial and domestic uses. Therefore, like artificial reservoirs, these lakes are regulated primarily as storages. A comparison of our results with previous studies^12^,^26^ shows that, of 97,019 sluice gates with capacity greater than 1.6 × 10^8^ m^3^, approximately 60% are located in southeast China, especially in the middle and lower Yangtze reaches. By combining the addresses of sluice gates^26^ with lake locations, we conservatively estimated that 70% of freshwater lakes with area greater than 10 km^2^ are regulated. This phenomenon is more widespread in the middle and lower Yangtze reaches–almost all the large lakes are regulated except the Dongting and Poyang Lakes^27^.

### Possible causes of lake disappearance

With respect to the five geographic lake regions, the changes show two important trends (Figure 5). First, in the TPL, the number of lakes of ≥1 km^2^ shows an increase of 49 lakes and the total surface area also slightly increased accordingly; secondly, in other four lake regions, both lake number and surface area decreased by varying degrees. The greatest reduction occurred in the IMXL, with a loss of approximately 9,500 km^2^, or ~50% of lake area resulted from approximately vanished lakes and many shrunken lakes; the second largest decrease occurred in the Eastern Plain Lake Region (EPL) which lost 95 large lakes representing a surface area of 7,025.7 km^2^. Much of this reduction occurred in the middle and lower reaches of the Yangtze River Basin and the Huaihe River Basin.

However, the causes of the uneven spatial distribution of changes in lake number and lake area vary dramatically in different lake regions. Some researchers have reported that the reduction in lake in IMXL has been caused by climate change^28^, because this area, for example, the Tarim River Basin, characterized by an arid climate, has observed a warming trend beginning in the 1950s. Shi et al.^29^ reported that, since 1980s, air temperature and precipitation in the northwest China, including the Tarim River Basin, has been increasing rapidly. The increasing trend is more significant in Northwest China than in East China. Shi et al.^29^,^30^ even concluded the strong signals of climatic shift to warm humid pattern have been appearing in the Northwest China. However, contrary to the increase in precipitation, the water discharge at most hydrological stations of the Tarim River decreased significantly: anthropogenic impacts, such as soared water consumption by irrigation and damming of rivers have substantially reduced the water discharged into lakes at an increasing rate^10^. The Tarim River Basin has rapid population growth, accelerated reclamation and large irrigation diversions in the basin. The population has increased rapidly from about 3 × 10^6^ in 1949 to 8.5 × 10^6^ in 2000^31^ and irrigated land jumped 2.2 fold in this period. By 2005, more than one-third of main-stem flow was withdrawn for irrigation, leading to sharp decrease in water level^32^. Therefore, although the disappearance of lakes happened in the context of global climate change, it principally reflects the severe anthropogenic impacts in these areas.

In the EPL, the key causes are land reclamation and lake isolation. For example, in the late 1940s, lakes covered a total area of about 35,123 km^2^ in the middle and lower reaches of the Yangtze River^9^. About 12,000 km^2^ of lake area were drained for farming from the 1950s to the 1970s^33^. In many cases, whole lakes were drained. The Dongting and Poyang lakes are the two key examples showing the rapid decrease in lake surface area since the 1950s. The surface of the Dongting Lake has decreased by 37%, from 2,825 km^2^ in 1950s to 1,785 km^2^ in 2008 primarily due to anthropogenic activity, such as littoral land reclamation. The area of the Poyang Lake has also decreased significantly as a result of land reclamation^34^. Total reclaimed land from1949 to 2007 measured 2,300 km^2^ and resulted in a decrease in surface lake area from 5,200 km^2^ to 2,900 km^2^, indicating a 45% decrease in the sixty-year period.

In the Tibet Plateau both surface area and the number of lakes increased. The changes could also reflect climate change over the past decades, with the areas at altitudes above 4000 m having warmed 0.3°C per decade^35^. Song et al.^36^ show that the variations in different lakes on the Tibet Plateau are dependent on their own complex hydrological and climatic environment in their lake basins because many factors influence the water budget of lakes, such as, precipitation, snow melt, underground water and evaporation. Thus, the dominant factor varies greatly from lake to lake. For example, the evaporation rate in some areas at elevation of 2,800–4,800 m is two to three times higher than the TP's precipitation rate^37^. The large negative budget caused by “precipitation-evaporation” has resulted in dramatic lake shrinkage despite the slight increase in precipitation^38^. For instance, Song et al.^36^ indicated that Lake Qinghai on the northeastern Tibet, which is supplied primarily by rainfall runoff, was shrinking until the early 2000s because of the rising air temperature. Li et al.^39^ also reported that the variation of water level of the Qinghai Lake was highly positively correlated to surface runoff and precipitation and negatively to evaporation, the correlation coefficients were 0.89, 0.81 and −0.66, respectively. The study by Li et al.^39^ indicated that precipitation-evaporation negative budget is the dominant factors for the decline in water level of the Qinghai Lake.

In contrast, meltwater from mountainous glaciers and snow cover have become important water sources for lakes at relatively high altitudes (elevation > 4,800 m), a conclusion that is also supported by the study of Zhang et al.^19^. Of all the 261 lakes with surface greater than 10 km^2^, 131 lakes underwent expansion; only 56 lakes underwent shrinkage. When we investigated these lakes at different elevation ranges, some evident trends appeared: only 30% of shrunken lakes are located at range of 4,800–5,400 m which means that most of the shrunken lakes are located at relatively low altitudes (<4,800 m); 52% of enlarged lakes are located at range of 4,800 m–5,400 m, indicating that enlarged lakes are prone to occurring at relatively high altitudes. For example, by studying Nam Co Lake, the highest lake in the central Tibetan Plateau (~4,800 m), Zhang et al.^40^ and Phan et al.^41^ reported that the enlarging status of Nam Co Lake water storage is closely related to increasing of stream runoff especially coming from the input of glacial meltwater. Consequently, it can be concluded that lakes at relatively low altitudes (28,00–4,800 m) are particularly prone to shrinking negative precipitation-evaporation budget caused by climate warming; while the counterparts at relatively high altitudes (>4,800 m) are more likely to expand due to increasing water supply from glaciers caused by climate warming. With the increasingly intensive climate warming tendency, the large amount of meltwater from mountainous glaciers/snow and subsurface permafrost has caused these lakes to expand rapidly.

### The degree of river regulation

The proportion of a river's annual runoff that can be withheld by a reservoir or a cluster of reservoirs can serve as a first-level approximation of the potential impact on downstream flows^5^,^7^. This index, which – following other authors – we term “degree of regulation (DOR)”, has in one form or another, been a key component of seminal studies on flow regulation^5^,^7^. DOR is defined as the ratio between total reservoir storage capacity and annual average runoff. A high DOR value indicates an increased probability that substantial discharge values can be stored throughout a given year and released at later times. Both temporal storage and delayed release alter the natural flow regime and, as a result of the increased stagnation and stratification of the stored water, can also affect other characteristics such as sediment load and flow velocity.

Figure 6 shows the affected large rivers and their major tributaries by water regulation. Dynesius and Nilsson^42^ used a DOR threshold of 2% - equivalent to the capacity of storing about one week of the total annual flow – to distinguish between free-flowing rivers and the onset of environmental consequences. In the same sense, here we refer to rivers with a DOR ≥ 2% as “affected” rivers. We also provide additional results for a suite of higher DOR thresholds in order to support a more differentiated interpretation in Figure 6. Adopting a DOR threshold of 2%, we find that only the Salween and Yarlong rivers, or only 6% of the assessed river basins, are free-flowing. Nilsson et al.^7^ classified the river systems with dams constructed on mainstem or major tributaries into three levels of impact: strongly affected (DOR > 30%), moderately affected (2% ≤ DOR ≤ 30%), and not affected (DOR < 2%). Based on this rule, all the rivers in Table 3 with ratio of capacity to runoff (DOR) greater than 0.3 (30%) are strongly affected except the Yangtze River. However, Nilsson et al.^7^ also indicated that if a river like the Yangtze (with DOR > 15%) has two or more dams (the Gezhouba and Three Gorges dams) on mainstem, the river should also be considered as strongly affected. This means that almost all the large outflow rivers are strongly affected by reservoirs in China. This is an alarming result compared to the global average of only 7.6% of the world's rivers with average flows above 1 cubic meter per second (m^3^ s^−1^) affected by reservoirs^5^. In China, 129 × 10^4^ km^2^, or 20% of assessed river basins have enough cumulative reservoir capacity in their respective upstream catchment to store more than the entire annual river flow (DOR ≥ 100%). All of these rivers are located in North and Northeast China, such as the Liaohe River (367%), the lower Yellow River (171%), the major tributaries of the Songhua River (165%), the Huaihe River (120%) and the Haihe River (109%). Rivers in South and Southwest China, such as the Yangtze and Pearl rivers, have relatively small DOR values. Although smaller DOR ratios may imply less of a general impact, some critical aspects of the flow regime may still be strongly altered. For example, the Three Gorges Dam (TGD) and other large dams in the upper Yangtze River Basin can store only 36% of the total annual flow of the upper Yangtze River, but these reservoirs, especially the TGD, has substantially altered the downstream sediment transport of the Yangtze River^18^.

The DOR values in different sub-basins of Chinese rivers can be significantly different. For example, the DOR ratio of the Min River in the upper Yangtze River Basin is relatively small (8%), but the DOR value for the Wu River in the upper Yangtze River Basin is as high as about 68% because the Wu River is fully regulated by 9 cascade dams. Other tributaries such as the Jialing and Han rivers in the Yangtze River Basin also stand out as being highly affected resulting from a multitude of dispersed reservoirs. In other basins such as the Yellow River Basin and the Pearl River Basin, effects are concentrated to certain sub-basins as well.

Singular reservoirs can have the potential for abrupt but severe alterations in the DOR ratio, and the effects can propagate far downstream on the main-stem river, as is apparent in the Yangtze River (e.g. TGD and the Gezhouba reservoir), Yellow River (e.g. the Sanmenxia and the Xiaolangdi reservoirs), Pearl River (e.g. the Longtan and Yantan reservoirs) and the Songhua River (e.g. Fengman, Baishan and Ni'erji reservoirs). Our assessment results indicate that more than 90% of large Chinese river systems are affected by dams and rivers in North China have higher DOR values than those in South China.

## Conclusions

The integration of Landsat TM/ETM+ images allows for understanding anthropogenic impacts on waterscape change in a large-scale area. In this study reported herein, we employ satellite images and other auxiliary data to enable the delineation of lakes and reservoirs across the mainland China and comprehensive estimates of changes in lake and reservoir storage capacity using statistical models. The results provide comprehensive information on lake and reservoir changes in China. Based on the results, this study also provides valuable assessment on the resultant river fragmentation.

The study results show that dramatic changes have occurred to the reservoirs and lakes in mainland China over the past 60 years. Numerous reservoirs with a total storage capacity of 794 km^3^ with a total surface area of 26,870 km^2^ have appeared, but 350 lakes greater than 1 km^2^ with a total surface area of 18,151 km^2^ have disappeared. Anthropogenic activities, such as, excessive water consumption in the IMXL, land reclamation and urbanization in the EPL are the causes of the disappearance of nearly 350 large lakes in mainland China. The study also shows that almost all the large rivers are significantly affected by reservoirs in China, only 6% of which are free-flowing. Approximately 20% of assessed river basins have enough cumulative reservoir capacity in their respective upstream catchment to store more than the entire annual river flow. All of these rivers are located in North and Northeast China.

## Methods

### Data sources and data preprocessing

**Figure 1:**
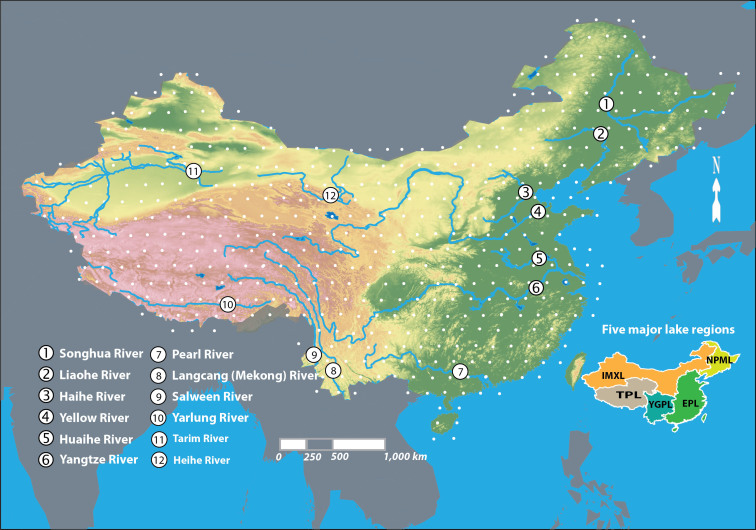
The Landsat TM/ETM+ path and row coordinates (white points) defining the conterminous mainland China. The figure also highlights 12 large rivers which are one of the highlights of this study. The inset shows the five geographic lake regions in China. These are (1) the Tibetan Plateau Lake Region (TPL), covering Qinghai Province and Tibet Autonomous Region; (2) the Yunnan-Guizhou Plateau Lake Region (YGPL), covering Yunnan, Guizhou and Sichuan provinces and Chongqing Municipality; (3) the Inner Mongolia-Xinjiang Lake Region (IMXL), covering Inner Mongolia, Xinjiang Uygur and Ningxia Hui autonomous regions, and Gansu, Shaanxi and Shanxi provinces; (4) the Northeast Plain and Mountain Lake Region (NPML), covering Liaoning, Jilin and Heilongjiang provinces; and (5) the Eastern Plain Lake Region (EPL), covering Shanghai, Beijing and Tianjin Municipalities, Hong Kong and Macao special administrative regions, and Jiangxi, Hunan, Hubei, Anhui, Henan, Shandong, Zhejiang, Jiangsu, Hainan, Fujian, Guangdong and Guangxi provinces. Rivers 1–10 are exorheic rivers; rivers 11–12 (Tarim and Heihe rivers) are inland rivers. The figure was created using ESRI ArcGIS 9.3. The DEM data used in this figure was download from the Consortium for Spatial Information of the Consultative Group on International Agricultural Research^8^.

**Figure 2:**
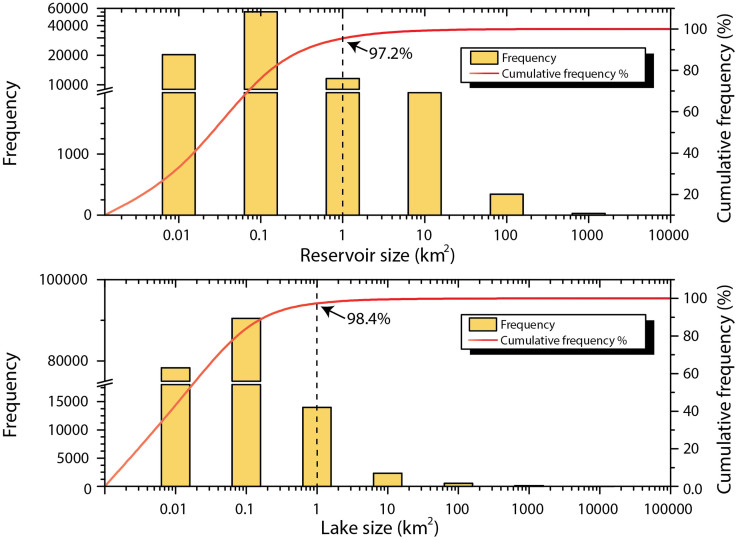
Cumulative frequency of number and area of lakes and reservoirs. The dot line indicates that most lakes and reservoirs are small (<1 km^2^): 97.2% of reservoirs and 98.4% of lakes are less than 1 km^2^ in surface area.

**Figure 3:**
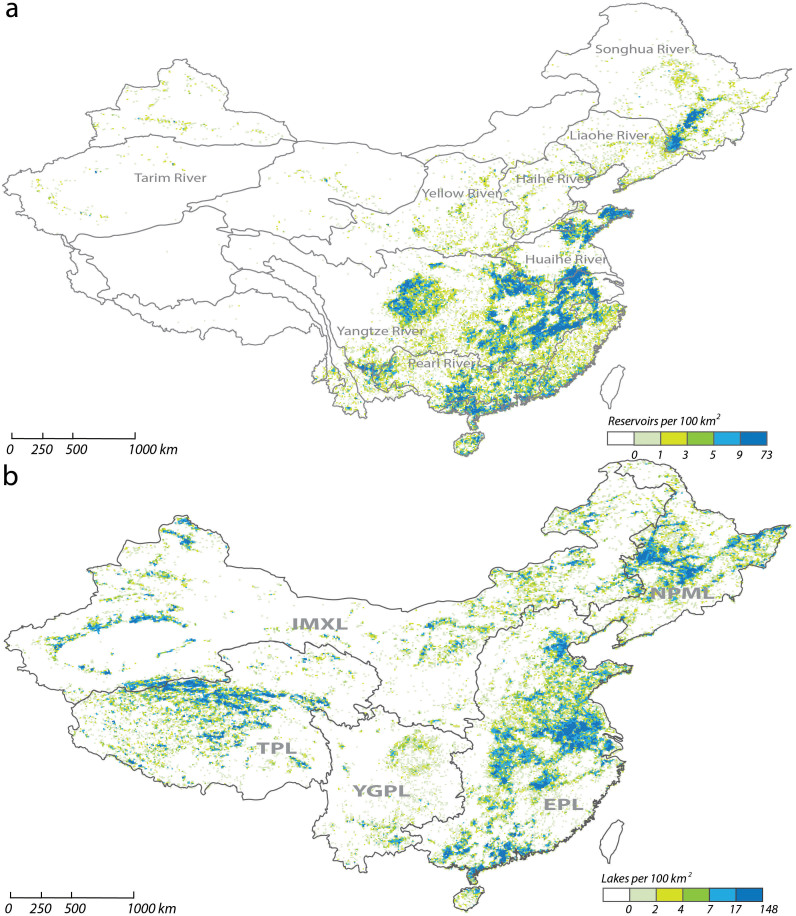
The variation of the density of reservoirs (a) and lakes (b) across mainland China. The upper panel shows a clear east-to-west gradation in the spatial distribution of reservoirs: most of the reservoirs are located in the eastern regions such as the middle and lower Yangtze River Basin, the lower Pearl River Basin and some small river basins in Southeast China. The lower panel shows the uneven distribution of lakes in China: 50% of lake area in TPL, 25% in the EPL, 15% in the IMXL, 8.5% in the NPML and 1.5% in the YGPL. The map was created using ESRI ArcGIS 9.3.

**Figure 4:**
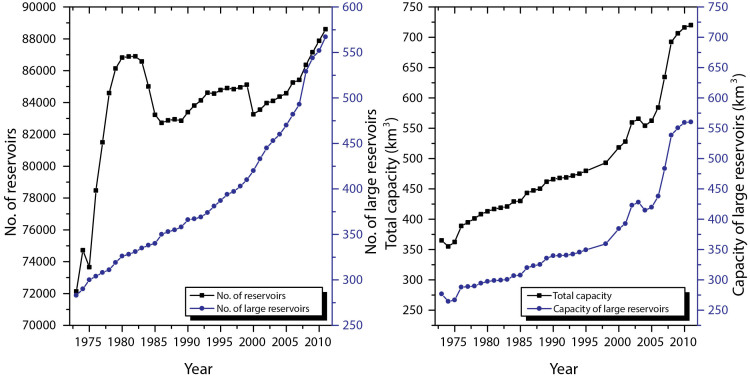
History of increase in number and total storage of all reservoirs (black dotted line) and large reservoirs (blue dotted line) in mainland China. The number fluctuation in the 1980s and 1990s shows that some reservoirs built during the period of the 1950s ~ 1970s were in poor quality, which have been scrapped in these periods due to ageing and lack of proper maintenance.

**Figure 5:**
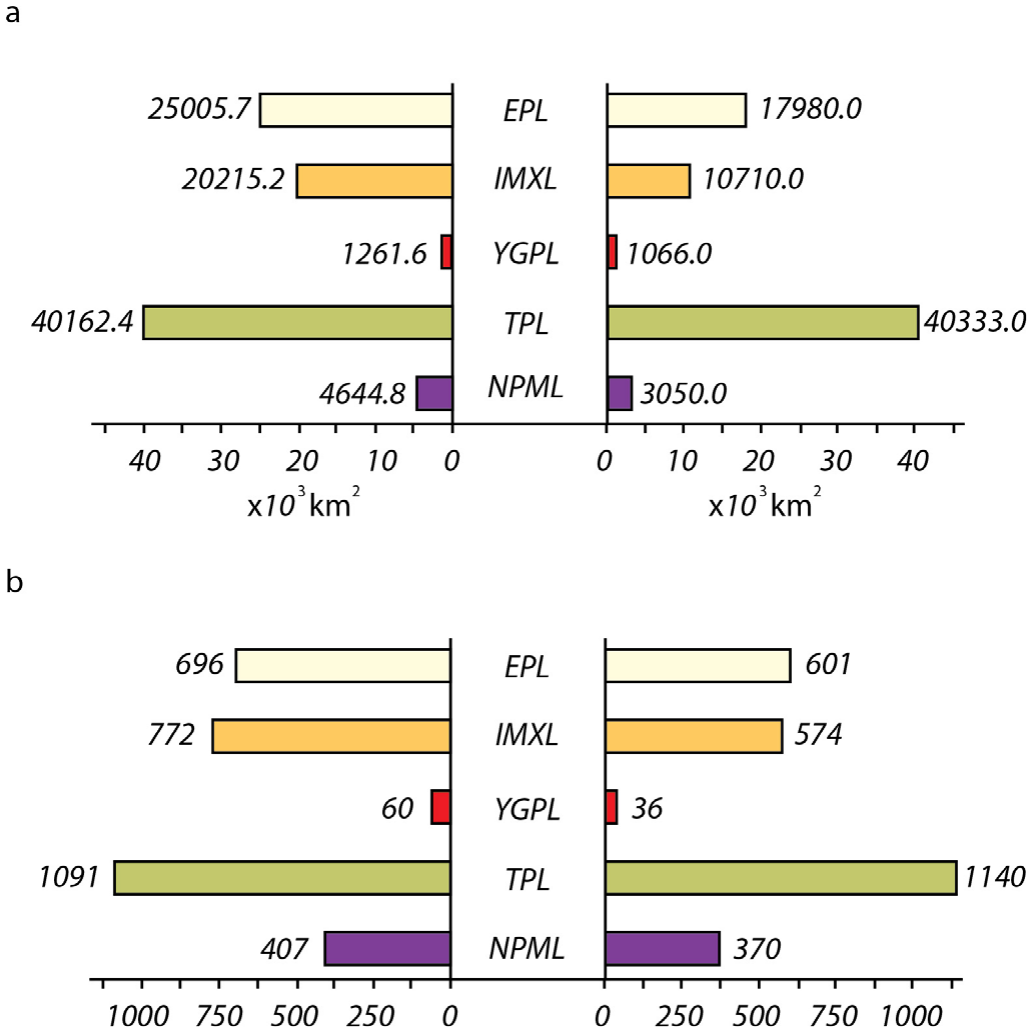
The contrasts of number and surface area of lakes (≥1 km^2^) between our results and data documented by Wang and Dou^8^. (a) Changes in lake area in the five lake regions; (b) changes in lake number in the five lake regions. Data on the left side were mainly collected from the 1950s to 1970s^8^ and updated in the 1980s (not shown in the figure); data on the right side are the results of this study.

**Figure 6:**
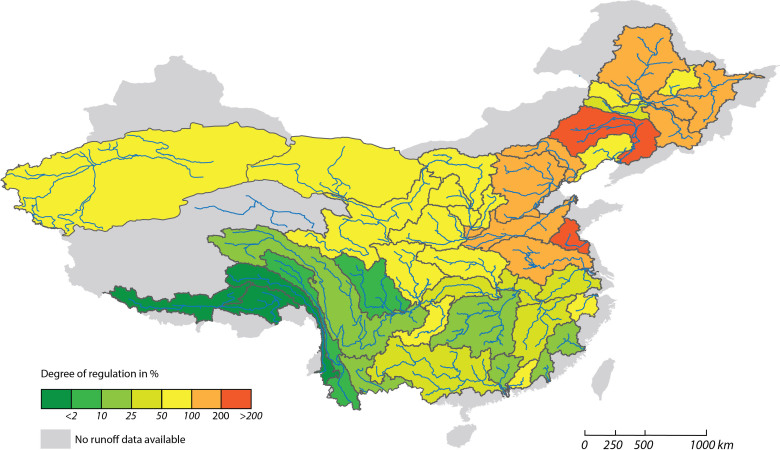
The affected large rivers and their major tributaries by water regulation. Only 6% of the assessed river basins are free-flowing; 20% of assessed river basins have enough cumulative reservoir capacity in their respective upstream catchment to store more than the entire annual river flow. The map was created using ESRI ArcGIS 9.3.

Remotely-sensed data provide a means of delineating water body boundaries over a large area at a given point in time. The Landsat program is the longest running enterprise for acquisition of satellite imagery of Earth. Its Landsat Thematic Mapper (TM) and Enhanced Thematic Mapper Plus (ETM+), which acquired digital-format imagery with 30 m spatial resolution in seven spectral channels, have become a unique resource in the study of albedo and its relationship to global warming and water scape change. Landsat TM/ETM+ images, mainly acquired after the monsoon season (September–October) in the period 2005 to 2008, were used in this study. A total of 507 images, including 412 TM images and 95 ETM+ images, were used (Figure 1). On 31 May 2003, the ETM+ Scan Line Corrector (SLC) failed, causing the scanning pattern to exhibit wedge-shaped scan-to-scan gaps. Images acquired after the SLC failure are referred to as SLC-off images. In this study only SLC-off images were used. An approach^43^ was used to fill gaps in Landsat ETM+ SLC-off images. Ideally, contemporary data for the same year were used, but the limited availability of cloud-free data necessitated the use of data from multiple years (from 2005–2008). Even then, we could not find all cloud-free images covering the entire mainland China. Haze correction and cloud removal for some images was used in image pre-processing. 17 images with clouds were used. For images with thin clouds or hazes on images, the approach proposed by Martinuzzi et al.^44^ was used for haze correction. For images with thick clouds, we have developed a program based on thresholding that segments an image into two categories (cloud, non-cloud) defined by a single DN (digital number) threshold. The detected thick clouds were then replaced with a suitable value by looking into next or previous available images. After that, the images could be used in this study.

The overall procedure of image processing can be summarized into two phases, namely, water body detection and water body classification. Because processing approximately 500 satellite images would have been time-consuming and labor-intensive, we developed an automated procedure that employs multiple thresholds, generating various DN magnitudes, such as normalized difference vegetation index (NDVI)^45^ and normalized difference water index (NDWI)^46^ and differences in the spectral characteristics of different land cover types (e.g., water, snow, bare land and vegetation) in visible, near infrared and mid-infrared bands. The normalized difference snow index (NDSI)^47^ threshold was also used to remove the impact of snow on Tibetan Plateau. Also, Digital Elevation Model (DEM) data were integrated in this program to remove the impact of shadows in mountainous areas. However, it should be noted that no specific thresholds for the parameters were set because our researchers could manually adjust the threshold to achieve the best overall result for each image. In this step, satellite images were classified into two categories: water and non-water. The results were then converted into polygons with contiguous pixels and stored in a shapefile. Subsequently, as a result of filtering, any object smaller than 4 pixels or 0.0036 km^2^ was automatically removed from the data to remove image noises. The removed water bodies are insignificant in size; therefore, they had a negligible effect on the total area.

In the second step, based on secondary data and high-resolution satellite data from the Google Earth, the polygons were visually interpreted to classify water bodies into three main classes: lakes (lakes and ponds), artificial reservoirs and rivers. One of the major impediments to the classification was that, there are numerous paddy fields and aquacultural farms which have similar spectral characteristics to natural lakes in the lower reaches of the Yangtze and Pearl rivers. To reduce misclassification error, we used ancillary data, visual interpretation and expert knowledge of the area through GIS to visually interpret the images. Using visual cues, such as tone, texture, shape, pattern, and relationship to other objects (such as a reservoir is often associated a dam), an observer can identify many features on a high-resolution image (such as Google Earth imagery). We have also developed a computer program to assist our researchers visually classify each polygon into different water-body types. Researchers could easily and efficiently classify each polygon into different water-body types. After classification, other features of the water bodies such as surface area, names and administrative divisions were also added to the dataset.

### Estimating reservoir and lake storage capacity

Many researchers have demonstrated the existence of a robust relationship between the surface area and volume capacity of lakes and reservoirs at both regional and global scales^4^,^5^,^11^. This relationship was used to develop a method for area-based estimation of reservoir storage capacities. Meigh^48^ first introduced a formula for the power relationship between capacity (*C*; 10^6^ m^3^) of a reservoir and its surface area (*A*; km^2^): 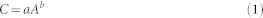
Where *a* and *b* are constants.

To build the regression equation, data on the storage capacities of 2,185 reservoirs (0.01–10 km^3^) and 1,118 large lakes (surface area ≥ 1 km^2^) were collected from official documents of the Chinese government, particularly a series of reports on reservoir development. A number of other ancillary data sources as well as information from previous studies^9^,^49^,^50^ were also used. We adopted a very conservative approach to data collection; i.e., only storage capacity values that appeared in multiple sources were used in order to guarantee data quality. Although we selected only 2,185 reservoirs, most of the reservoirs are large or medium reservoirs, the cumulative capacity is approximately 514 km^3^, or 65% of the total estimated reservoir capacity. Likewise, the cumulative storage volume of selected lakes is approximately 189 km^3^, or 72% of the total estimated lake storage volume. In addition, we selected the lakes and reservoirs primarily based on the size (capacity for reservoirs, surface area for lakes); thus, the selected data represent similar spatial distribution to the entire water bodies in mainland China. The selected lakes and reservoirs can be a reasonable representative of the whole China's lakes and reservoirs.

The equations established for reservoirs and lakes were as follows: 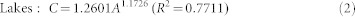

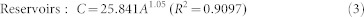
Where *C* is reservoir storage volume for individual reservoirs or reservoirs in 10^6^ m^3^, and *A* is the surface area in km^2^.

The deviation area index (DAI) was therefore used to quantify the difference between the surface area derived from Landsat TM/ETM+ images and the area delineated in high resolution images provided by Google Earth in the similar period. Because image acquisition mainly took place in 2005 and 2008 and there was no field work during this period, there was no possible comparison between water body found in the field and in the images. The assessment result was presented in Supplementary document.

## Author Contributions

X.X. conceived the idea. X.K. delineated and classified water bodies in the remote sensing images; X.X. helped to design the methods of this study; X.K. performed validation statistical analysis of delineation results; X.K. prepared the figures and wrote the manuscript, which subsequently revised by X.X.

## Supplementary Material

Supplementary Information Supplementary document

## Figures and Tables

**Table 1:** The numbers, average sizes, and areas of lakes delineated from satellite images

Size range (km^2^)	No. of lakes	Average area (km^2^)	Total area (km^2^)	Total capacity (km^3^)
0.0036–0.01	78,163	0.0069	540	0.3
0.01–0.1	90,451	0.028	2,556	1.8
0.1–1	13,998	0.27	3,796	4
1–10	2,060	2.9	5,974	11
10–100	533	31	16,610	40
100–1000	118	264	31,098	107
1000–10000	10	2166	21,658	104
Total	185,333	--	82,232	268.1

**Table 2:** The numbers, average sizes, and areas of reservoirs delineated from satellite images

Size range (km^2^)	No. of reservoirs	Average area (km^2^)	Total area (km^2^)	Total capacity (km^3^)
0.0036–0.01	20,507	0.0070	144	3
0.01–0.1	55,188	0.031	1,687	37
0.1–1	11,500	0.29	3,330	83
1–10	2,120	2.7	5,819	162
10–100	354	27	9,421	276
100–1000	27	240	6,470	233
Total	89,696		26,870	794

**Table 3:** General characteristics, capacity–area and capacity–runoff ratios for large rivers in China

River	Drainage area (10^4^ km^2^)	Runoff (km^3^ yr^−1^)	No. of reservoirs	Reservoir capacity (km^3^)	Capacity/area (10^3^ m^3^ km^−2^)	Capacity/runoff (yr)
Yangtze River	180	951.3	43,602	262 (286.3)^b^	146	0.28
Songhua River	84.7	116.6	5,016	103 (83)	122	0.88
Liaohe River	29.7	14.8	1,309	38 (39.3)	128	2.57
Haihe River	29.2	22.8	1,041	33 (32)	113	1.45
Yellow River	78	20.7^a^	2,747	65 (82.7)	83	3.14
Huaihe River	31.2	62.2	9,806	58 (62)	186	0.93
Pearl River	56.3	333.8	17,016	106 (109.6)	188	0.32
Other rivers in Southeast	25.7		6,295	56 (56.7)	218	
Other rivers in Southwest	144.9		1,897	57 (42.5)	39	
Other rivers in Northwest	248.9		967	16 (15.9)	6	
Total	908.6		89,696	794 (810)	87	

^a^The runoff of the Yellow River has reduced dramatically since the 1960s; the runoff value used here is the mean runoff from 1976 to 2008.

^b^Data from the Ministry of Water Resources (MWR)^11^.

**Table 4:** Number and area of lakes delineated on remote-sensing imagery in different river basins

River basin	Drainage area(10^4^ km^2^)	No. of lakes	Total lake area (km^2^)	Percent water (%)	Lakes per 100 km^2^	No. of lakes (≥1 km^2^)
Yangtze River	180	43,185	16,579	0.92	2.40	577
Songhua River	84.7	24,440	8,109	0.96	2.89	441
Liaohe River	29.7	4,188	403	0.14	1.41	55
Haihe River	29.2	6,327	285	0.10	2.17	29
Yellow River	78	8,677	6,490	0.83	1.11	136
Huaihe River	31.2	22,605	4,983	1.60	7.25	86
Pearl River	56.3	22,182	766	0.14	3.94	14
Rivers in Southeast	25.7	2,864	128	0.05	1.11	10
Rivers in Southwest	87	5,213	3,005	0.35	0.60	38
Rivers in Northwest	248.9	21,622	10,077	0.40	0.87	510
Tibetan inland rivers	65.6	24,030	31,407	4.79	3.66	825
Total	916.3	185,333	82,232	0.90	2.02	2,721

## References

[b1] Macdonald G. M. *et al.* Rapid Response of Treeline Vegetation and Lakes to Past Climate Warming. Nature 361, 243–246 (1993).

[b2] Tranvik L. J. *et al.* Lakes and reservoirs as regulators of carbon cycling and climate. Limnol Oceanogr 54, 2298–2314 (2009).

[b3] Williamson C. E. *et al.* Lakes and reservoirs as sentinels, integrators, and regulators of climate change. Limnol Oceanogr 54, 2273–2282 (2009).

[b4] Downing J. A. *et al.* The global abundance and size distribution of lakes, ponds, and impoundments. Limnol Oceanogr 51, 2388–2397 (2006).

[b5] Lehner B. *et al.* High-resolution mapping of the world's reservoirs and dams for sustainable river-flow management. Front Ecol Environ 9, 494–502 (2011).

[b6] Smith S. V. *et al.* Distribution and significance of small, artificial water bodies across the United States landscape. Sci Total Environ 299, 21–36 (2002). 12462572 10.1016/s0048-9697(02)00222-x

[b7] Nilsson C. *et al.* Fragmentation and flow regulation of the world's large river systems. Science 308, 405–408 (2005). 15831757 10.1126/science.1107887

[b8] Jarvis A. *et al.* Hole-filled seamless SRTM data V4. International Centre for Tropical Agriculture (CIAT) (available from http://srtm.csi.cgiar.org) (2008).

[b9] Wang S. M. & Dou H. S. Chinese Lake Catalogue (Science Press, Beijing, 1998).

[b10] Ma R. H. *et al.* A half-century of changes in China's lakes: Global warming or human influence? Geophys Res Lett 37, L24106 (2010).

[b11] Yang X. K. & Lu X. X. Delineation of lakes and reservoirs in large river basins: an example of the Yangtze River Basin, China. Geomorphology 190, 92–102 (2013).

[b12] MWR. Bulletin of first national census for water (China WaterPower Press, Beijing, 2013).

[b13] Lehner B. & Doll P. Development and validation of a global database of lakes, reservoirs and wetlands. J Hydrol 296, 1–22 (2004).

[b14] Yu J. *et al.* Effects of water discharge and sediment load on evolution of modern Yellow River Delta, China, over the period from 1976 to 2009. Biogeosciences 8, 2427–2435 (2011).

[b15] Brown L. & Halweil B. China's water shortage could shake world food security. World watch 11–21 (1998). 12348868

[b16] Wang H. J. *et al.* Interannual and seasonal variation of the Huanghe (Yellow River) water discharge over the past 50 years: connections to impacts from ENSO events and dams. Global Planet Change 50, 212–225 (2006).

[b17] Yao Y. H. *et al.* Large-scale hydroelectric projects and mountain development on the upper Yangtze River. Mt Res Dev 26, 109–114 (2006).

[b18] Xu K. H. & Milliman J. D. Seasonal variations of sediment discharge from the Yangtze River before and after impoundment of the Three Gorges Dam. Geomorphology 104, 276–283 (2009).

[b19] Zhang S. R. *et al.* Recent changes of water discharge and sediment load in the Zhujiang (Pearl River) Basin, China. Global Planet Change 60, 365–380 (2008).

[b20] Lu X. X. & Higgitt D. L. Recent changes of sediment yield in the Upper Yangtze, China. Environ Manage 22, 697–709 (1998). 9680538 10.1007/s002679900140

[b21] Lu X. X., Ashmore P. & Wang J. F. Seasonal water discharge and sediment load changes in the Upper Yangtze, China. Mt Res Dev 23, 56–64 (2003).

[b22] Walling D. E. Human impact on land-ocean sediment transfer by the world's rivers. Geomorphology 79, 192–216 (2006).

[b23] MWR. China Water Statistical Yearbook (China Water Power Press, Beijing, 2009).

[b24] Graf W. L. Dam nation: A geographic census of American dams and their large-scale hydrologic impacts. Water Resour Res 35, 1305–1311 (1999).

[b25] Chang W. Y. B. Large Lakes of China. J Great Lakes Res 13, 235–249 (1987).

[b26] MWR. Standard of the People's Republic of China: Code for China sluice gate name (China Water Power Press, Beijing, 2001).

[b27] Liu X. Q. & Wang H. Z. Estimation of minimum area requirement of river-connected lakes for fish diversity conservation in the Yangtze River floodplain. Divers Distrib 16, 932–940 (2010).

[b28] Yang J. P. *et al.* Variations of precipitation and evaporation in North China in recent 40 years. J Arid Land Res Environ 17, 6–11 (2003).

[b29] Shi Y. F. *et al.* Discussion on the present climate change from warm-dry to warm-wet in northwest China. Quaternary Sciences 23, 152–164 (2003).

[b30] Shi Y. F., Shen Y. & Hu R. Preliminary Study on Signal, Impact and Foreground of Climatic Shift from Warm-Dry to Warm-Humid in Northwest China. J Glaciol Geocryo 24, 219–226 (2002).

[b31] Zhang J. *et al.* Restoring environmental flows and improving riparian ecosystem of Tarim River. J Arid Land 2, 43–50 (2010).

[b32] Chen Z. S. *et al.* Changes of runoff consumption and its human influence intensity in the mainstream of Tarim river. Acta Geographica Sinica 66, 89–98 (2011).

[b33] Fu C. Z. *et al.* Freshwater fish biodiversity in the Yangtze River basin of China: patterns, threats and conservation. Biodivers Conserv 12, 1649–1685 (2003).

[b34] Shankman D. & Liang Q. Landscape changes and Increasing flood frequency in China's Poyang Lake region. Prof Geogr 55, 434–445 (2003).

[b35] Xu B. Q. *et al.* Black soot and the survival of Tibetan glaciers. Proc Natl Acad Sci USA 106, 22114–22118 (2009). 19996173 10.1073/pnas.0910444106 PMC2790363

[b36] Song C. Q., Huang B. & Ke L. H. Modeling and analysis of lake water storage changes on the Tibetan Plateau using multi-mission satellite data. Remote Sens Environ 135, 25–35 (2013).

[b37] Xie H. *et al.* Warming and drying trends on the Tibetan Plateau (1971–2005). Theor Appl Climatol 101, 241–253 (2010).

[b38] Zhang G. Q. *et al.* Snow cover dynamics of four lake basins over Tibetan Plateau using time series MODIS data (2001-2010). Water Resour Res 48, W10529 (2012).

[b39] Li X. Y. *et al.* Lake-level change and water balance analysis at Lake Qinghai, west China during recent decades. Water Resour Manag 21, 1505–1516 (2007).

[b40] Zhang B. *et al.* Estimation and trend detection of water storage at Nam Co Lake, central Tibetan Plateau. J Hydrol 405, 161–170 (2011).

[b41] Phan V., Lindenbergh R. & Menenti M. Geometric dependency of Tibetan lakes on glacial runoff. Hydrol Earth Syst Sc 10, 4061–4077 (2013).

[b42] Dynesius M. & Nilsson C. Fragmentation and flow regulation of river systems in the northern 3rd of the world. Science 266, 753–762 (1994). 17730396 10.1126/science.266.5186.753

[b43] Scaramuzza P., Micijevic E. & Chander G. SLC gap-filled products phase one methodology (U.S. Geological Survey, 2004).

[b44] Martinuzzi S., Gould W. A. & González O. M. R. Creating Cloud-Free Landsat ETM+ Data Sets in Tropical Landscapes: Cloud and Cloud-Shadow Removal (United States Department of Agriculture, Rio Piedras, 2007).

[b45] Tucker C. J. Red and photographic infrared linear combinations for monitoring vegetation. Remote Sens Environ 8, 127–150 (1979).

[b46] Gao B. C. NDWI--a normalized difference water index for remote sensing of vegetation liquid water from space. Remote Sens Environ 58, 12 (1996).

[b47] Sidjak R. Glacier mapping of the Illecillewaet icefield, British Columbia, Canada, using Landsat TM and digital elevation data. Int J Remote Sens 20, 273–284 (1999).

[b48] Meigh J. The impact of small farm reservoirs on urban water supplies in Botswana. Nat Resour Forum 19, 71–83 (1995).

[b49] MWR. Standard of the People's Republic of China: Code for China Lake Name (China Water Power Press, Beijing, 1998).

[b50] ICOLD. World register of dams (International Commission on Large Dams, Paris, 2011).

