# Supplementary material for: Drastic change in China's lakes and reservoirs over the past decades
Source: Sci Rep. 2014 Aug 13;4:6041. doi: 10.1038/srep06041 (PMC4131217; doi:10.1038/srep06041)
Supplement: Supplementary Information — Supplementary document [file srep06041-s1.doc]

Subject areas

HYDROLOGY, ENVIRONMENTAL SCIENCES, CLIMATE-CHANGE IMPACTS, ANTHROPOGENIC IMPACTS

Drastic change in China’s lakes and reservoirs over the past decades

Xiankun Yang^1^, Xixi Lu^1,2,*^

^1^Geography Department, National University of Singapore, 117570, Singapore, ^2^Global Change and Watershed Management Center, Yunnan University of Finance and Economics, Kunming, Yunnan, 650221, China

*Corresponding author. Tel: +65 6516 6135; Fax: +65 6777 3091

E-mail address: geoluxx@nus.edu.sg (Xixi. Lu)

**Supplementary Information**

**1. Water body delineation**

We have developed a computer program (Figure S1) that employs multiple thresholds, generating various DN magnitudes, such as NDVI[^1^](#_ENREF_1) and NDWI[^2^](#_ENREF_2) and differences in the spectral characteristics of different land cover types. In addition, we used the NDSI[^3^](#_ENREF_3) threshold to remove the impact of snow on Tibetan Plateau. Also, we integrated DEM data to remove the impact of shadows in mountainous areas. However, it should be noted we did not set specific thresholds for the parameters because our researchers can manually adjust the threshold to achieve the best overall result for each image.


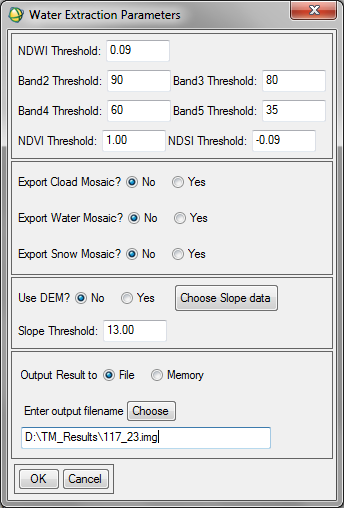


**Figure S1 |** A computer program developed by our researchers for water discrimination, the program was integrated into the software of ENVI 4.7.

The results were then converted into polygons with contiguous pixels and stored in a shapefile. One tricky problem for the shapefile was that many polygons overlapped mutually because the satellite images often overlap between adjacent scenes from a different path. In some areas there would be as much as 50% overlap between paths in China. To remove the overlaps the “Aggregate Polygons” tool within ESRI ArcGIS was used ([http://resources.arcgis.com/en/help/main/10.1/index.html#//00700000000s000000](http://resources.arcgis.com/en/help/main/10.1/index.html#//00700000000s000000)). The “Aggregate Polygons” tool is intended for moderate scale reduction and aggregation when input features can no longer be represented individually due to the limited map space or the required data resolution. It combines polygons within a specified distance of each other into new polygons (Fig. S2). Firstly, we set the distance to 0.0001 m, indicating only polygons which are very close to each other (less than 0.0001m) or overlap mutually would be combined into a new polygon (Step 1 in Fig. S2). All the overlaps were then eliminated accordingly. For some polygons close to the big ones, they actually belong to the big ones (circled part in Fig. S2), but are “dissected” into two parts by a bridge or other barriers. We again used the “Aggregate Polygons” tool with a specified distance of 10.0 m to combine the small polygons into the big one (Step 2 in Fig. S2) (the distance of 10.0 m in Fig. S2 is just an example; we adjusted this value in light of different situations).


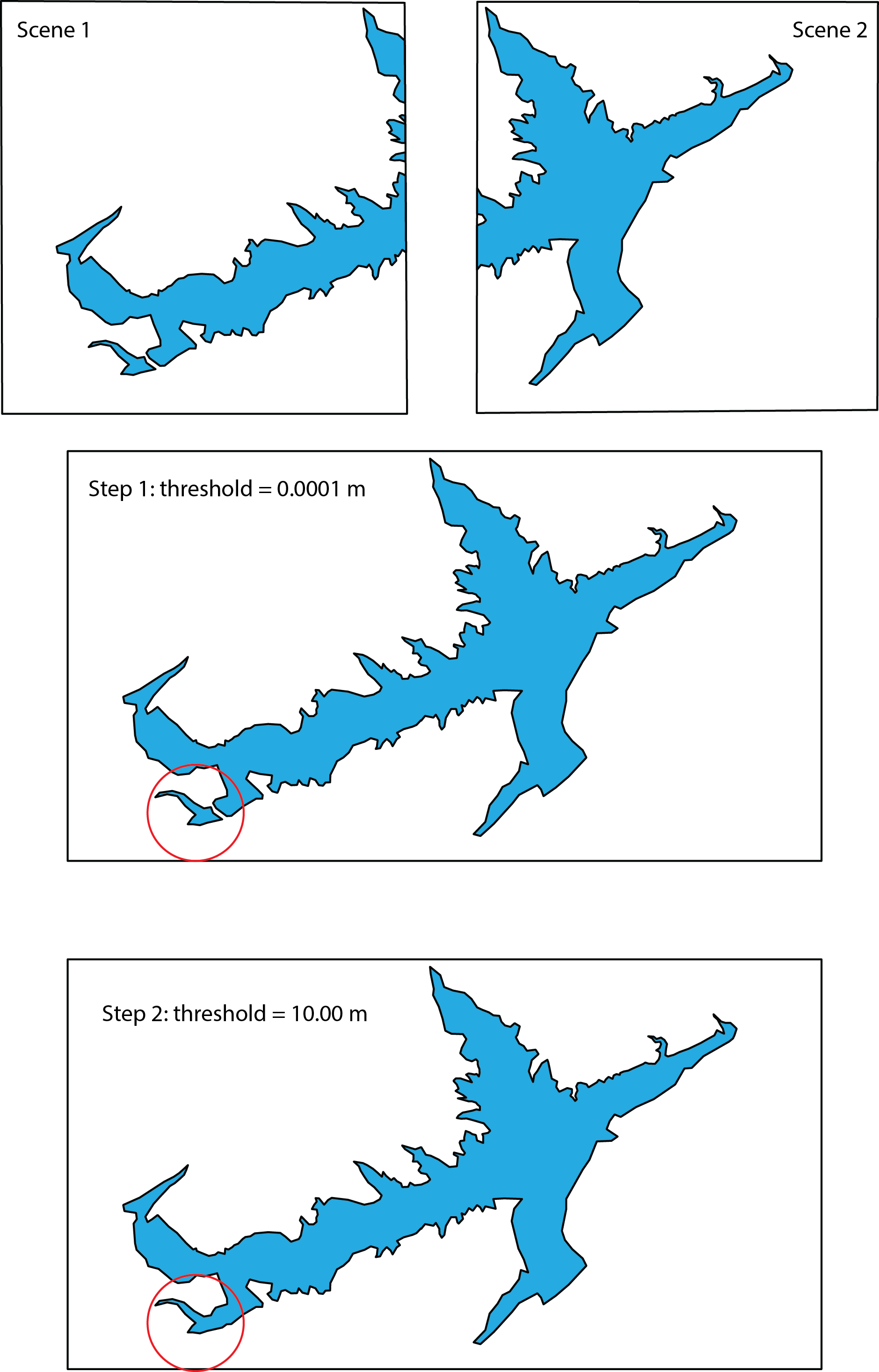


**Figure S2 |** An example of combination of polygons using the “Aggregate Polygons” tool within ESRI ArcGIS. The map was created using ESRI ArcGIS 9.3.

**2. Water body classification**

We used ancillary data, visual interpretation and expert knowledge of the area through GIS to visually interpret the images. The advantage of visual interpretation is the possibility to utilize contextual information and expert knowledge in the analysis more easily, sometimes in a more effective way, than in digital methods. We have developed a tool kit (Figure S3) to assist our researchers visually classify each polygon into different water-body types (lakes and ponds, reservoirs, and rivers). If one polygon is a paddy field, the polygon would be marked as deleted.


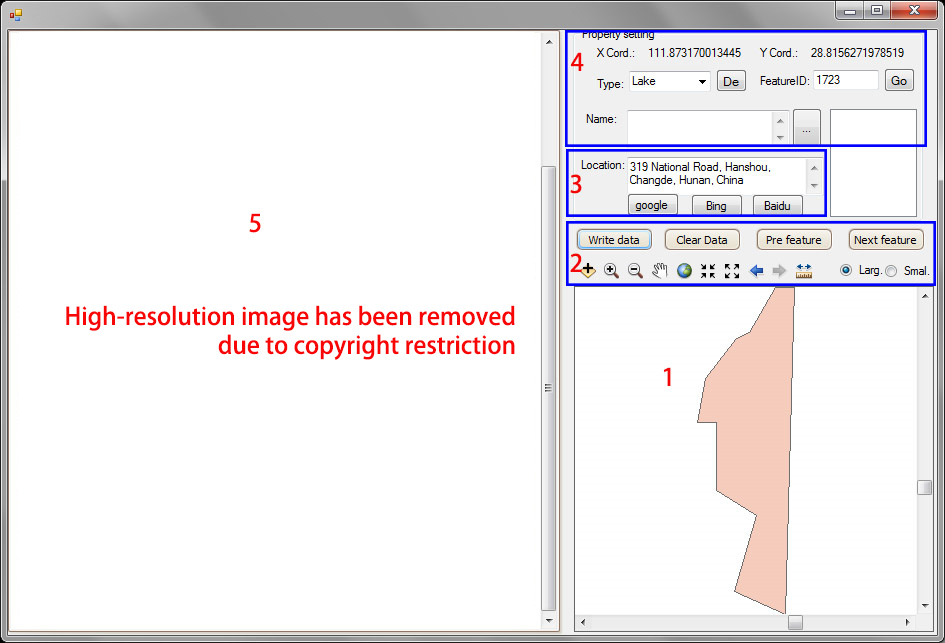


**Figure S3** | The tool kit used for visual interpretation. 1. Polygon to be classified; 2. Tools to operate map (zoom in, zoom out, pan etc.) (the “Write data” button is used to save information such as water type, location, name etc.); 3. Electronic maps/images (shown in left panel) used as auxiliary data; 4. Real-time data request from GeoNames geographical database based on the polygon’s coordinates. Using visual cues, such as tone, texture, shape, pattern, and relationship to other objects, our researchers could easily classify polygons into different types. The auxiliary data were automatically extracted by the tool kit, we could carry out the classification efficiently.

1. **Accuracy assessment of area estimates**

Because image acquisition mainly took place in 2005 and 2008 and there was no field work during this period, there was no possible comparison between water body found in the field and in the images. The deviation area index (DAI) was therefore used to quantify the difference between the surface area derived from Landsat TM/ETM+ images and the area delineated in high resolution images provided by Google Eearth^TM^ in the similar period. Nevertheless, it should be noted that the slight discrepancy between Landsat TM/ETM+ images and high-resolution images provide by Google Eearth^TM^ is an objective phenomenon due to different acquisition time. Thus, the method based on DAI is just rough assessment. The DAI is defined as follows:

$DAI=(Area_{G}-Area_{S})/Area_{G}$ (1)

where *Area_G_* is the surface area of lakes and reservoirs delineated in high resolution images using Google Earth^TM^ polygon tool; *Area_S_* is the surface area derived from Landsat TM/ETM+ images. The *DAI* values obtained from this equation would range from -∞ to 1. Water bodies with values close to zero have the best match between *Area_G_* and *Area_S_* while moving to extremes indicates increasing deviations between the two areas [^4^](#_ENREF_4).

In fact, manual digitizing on high-resolution images is extremely laborious, especially for large water bodies because the accuracy of manual digitizing merely depends on how accurate a water-body boundary is duplicated on a computer by hand. To get an accurate water-body boundary, one had to pick as many points as possible. 200 randomly selected water bodies with an area range of 0.05 km^2^—100 km^2^, including 100 reservoirs and 100 lakes were used to assess the accuracy. The 200 water bodies are randomly distributed in all the large river basins.


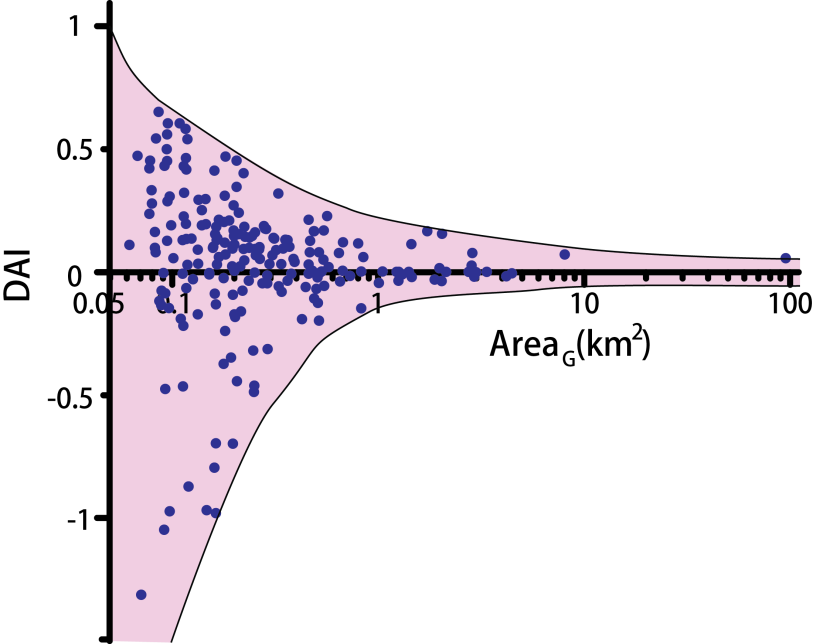


**Figure S4 |** DAI distribution against area of lakes and reservoirs delineated in high resolution images using Google Earth^TM^ polygon tool

The *DAI* distribution is shown in Figure S4. It can be seen most *DAI* values range between -0.3 and 0.3 and with the increase of the surface area, the absolute values of *DAI* close to zero, indicating that the larger are the water bodies, the better match of the surface areas. Figure 3 also shows that more DAI values for small water bodies are greater than zero, indicating small water bodies delineated in high-resolution images are slightly larger than their corresponding areas extracted from Landsat TM/ETM+ images. On average, the satellite based areas were found to be 8.1% smaller than the Google Earth^TM^ image based area estimates. This is not surprising because in Landsat TM/ETM+ images, reservoir and lake inlets could be identified until the width of the inlet/arm/peninsula is larger than 30 m due to the coarse resolution. This phenomenon was more common for small water bodies as it is more difficult to extract their boundaries on the coarse-resolution TM/ETM+ images. Although the accuracy for small water bodies (< 1 km^2^) is relatively low, lakes and reservoirs with area greater than 1 km^2^ contribute respectively 92% and 81% of the total surface area. Therefore, the relatively low accuracy for small water bodies has insignificant impact on further area-based analysis.

1. **Estimating reservoir and lake storage capacity**

This power relationship between the surface area and volume capacity of lakes and reservoirs was used to develop a method for area-based estimation of reservoir storage capacities. The established relationships are shown in Figures S5 and S6, respectively.


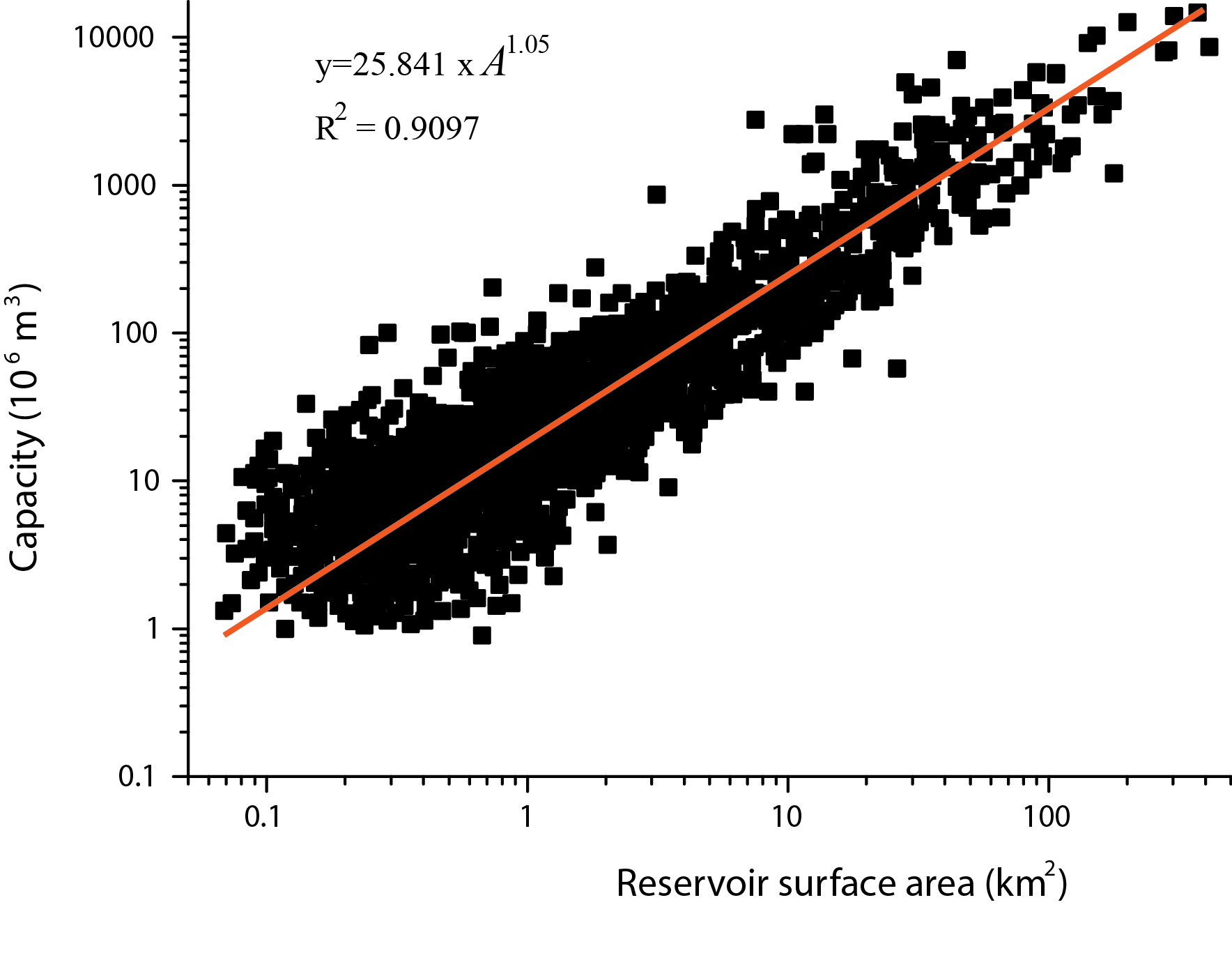
 **Figure S5 |** The correlation between capacities and their corresponding surface areas for reservoirs.


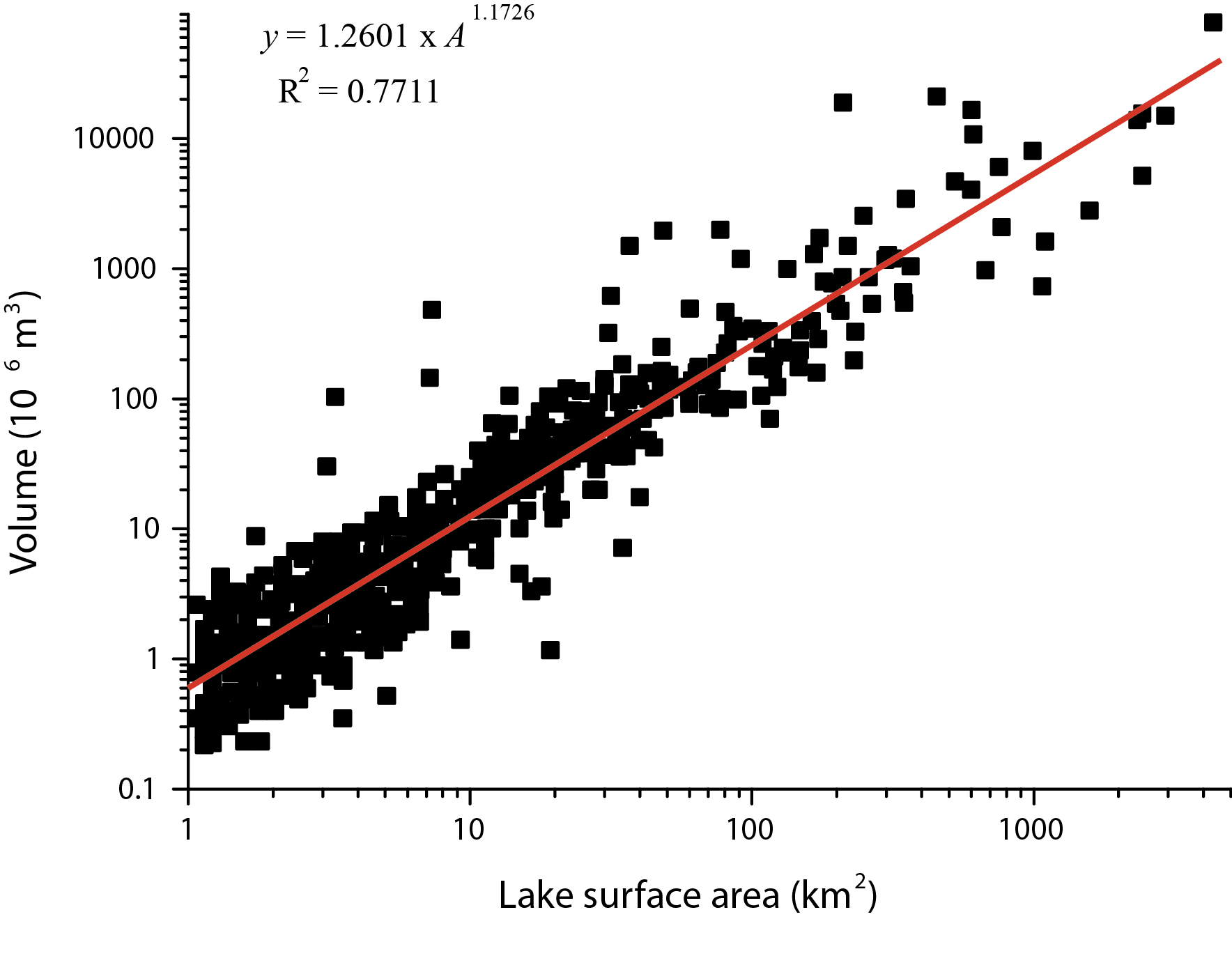


**Figure S6 |** The correlation between capacities and their corresponding surface areas for lakes.

The lake and reservoir capacities were calculated using the established power relationships. Further assessments were carried out based on the estimates.

**References**

1. Tucker, C. J. Red and photographic infrared linear combinations for monitoring vegetation. *Remote Sens Environ* **8:** 127-150 (1979).

2. Gao, B. NDWI--a normalized difference water index for remote sensing of vegetation liquid water from space. *Remote Sens Environ* **58:** 12 (1996).

3. Sidjak, R. Glacier mapping of the Illecillewaet icefield, British Columbia, Canada, using Landsat TM and digital elevation data. *Int J Remote Sens* **20:** 273-284 (1999).

4. Liebe, J., van de Giesen, N., Andreini, M. Estimation of small reservoir storage capacities in a semi-arid environment - A case study in the Upper East Region of Ghana. *PCE* **30:** 448-454 (2005).
